# Artificial Sweetener and the Risk of Adverse Pregnancy Outcomes: A Mendelian Randomization Study

**DOI:** 10.3390/nu16193366

**Published:** 2024-10-03

**Authors:** Di Mao, Mingmei Lin, Zhonghong Zeng, Dan Mo, Kai-Lun Hu, Rong Li

**Affiliations:** 1 Center for Reproductive Medicine, Department of Obstetrics and Gynecology, Peking University Third Hospital, Beijing 100191, China; 2110301326@stu.pku.edu.cn (D.M.); linmingmei2023@163.com (M.L.); zengbysy@163.com (Z.Z.); mo9919902@gmail.com (D.M.); 2 National Clinical Research Center for Obstetrics and Gynecology, Peking University Third Hospital, Beijing 100191, China; 3 Key Laboratory of Assisted Reproduction, Peking University, Ministry of Education, Beijing 100871, China; 4 Beijing Key Laboratory of Reproductive Endocrinology and Assisted Reproductive Technology, Beijing 100191, China; 5 Third Clinical Medical College, Peking University Health Science Center, Beijing 100083, China

**Keywords:** artificial sweetener, adverse pregnancy outcome, Mendelian randomization, ectopic pregnancy, premature rupture of membranes, placenta previa

## Abstract

The relationship between the intake of artificial sweetener (AS) and adverse pregnancy outcomes is under-researched, and existing studies yield inconsistent conclusions. A Mendelian randomization (MR) approach was employed to investigate the causal relationship between the intake of AS and adverse pregnancy outcomes. Instrumental variables related to the exposure phenotype were selected for analysis. The analysis was conducted using genome-wide association study summary data from public datasets. The inverse variance weighted, MR-Egger, weighted median, simple mode, and weighted mode methods were used to evaluate the causal relationship between exposure and outcomes. Sensitivity analysis and multivariable Mendelian randomization enrolling body mass index, type 2 diabetes mellitus, and fasting glucose were employed to further validate the consistency and robustness of the results. In univariable MR, the intake of AS added to tea was associated with an increased risk of ectopic pregnancy [OR = 1.821 (1.118–2.967), *p* = 0.016]. In multivariable MR adjusting for body mass index and type 2 diabetes mellitus, the intake of AS added to cereal was linked to a reduced risk of ectopic pregnancy [OR = 0.361 (0.145–0.895), *p* = 0.028] and premature rupture of membranes [OR = 0.116 (0.019–0.704), *p* = 0.019], while the intake of artificial sweetener added to coffee was associated with an increased risk of placenta previa [OR = 1.617 (1.042–2.510), *p* = 0.032]. No causal relationship was identified between the intake of artificial sweetener and other adverse pregnancy outcomes. The consumption of artificial sweetener during pregnancy warrants careful consideration.

## 1. Introduction

Artificial sweeteners (ASs), also referred to as non-nutritive sweeteners, were developed as substitutes for sugar. In comparison with conventional sweeteners such as sucrose and fructose, ASs are characterized by their high sweetness, low energy content, and good stability. Their ability to reduce sugar intake is believed to help prevent dental caries and obesity, making them a potential dietary option for patients with type 2 diabetes mellitus (T2DM). However, their effects on T2DM remain uncertain, with several studies indicating that AS intake was linked to increased risk of T2DM [1,2]. Currently, several common ASs, including aspartame, acesulfame K, saccharin, and sucralose, have received approval from various organizations, including the European Union and the United States Food and Drug Administration (FDA). These sweeteners are widely utilized in the production of everyday foods such as soda, bread, and pastries.

The safety of AS consumption during pregnancy has long been a subject of public health interest, with previous observational studies providing mixed results on its association with adverse pregnancy outcomes (APOs) such as low birth weight, preterm delivery, and gestational diabetes [3,4,5,6]. The fundamental constraints of observational studies, such as confounding variables and reverse causation, require a stronger methodological strategy to elucidate these relationships.

Mendelian randomization (MR) offers an alternative by using genetic variants as instrumental variables (IVs) under three core assumptions to assess causality in the relationship between exposures and outcomes. Our study aims to dissect these relationships further, providing evidence that may inform dietary recommendations for pregnant women and contribute to better pregnancy outcomes. 

## 2. Materials and Methods

### 2.1. Study Design

**Figure 1:**
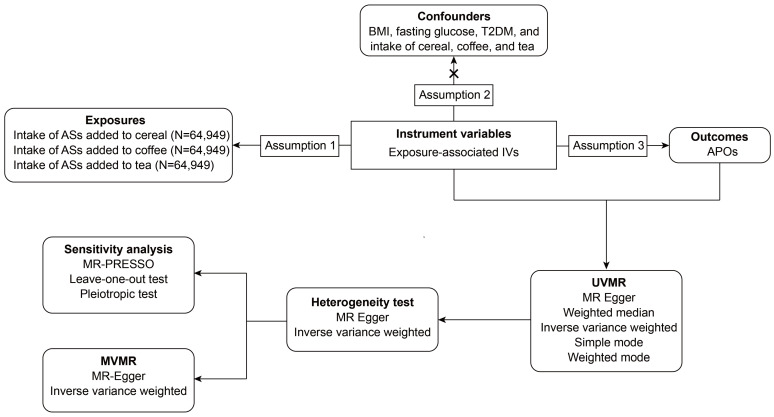
The overall flowchart of the two-sample Mendelian randomization study. BMI, body mass index; T2DM, type 2 diabetes mellitus.

A two-sample MR analysis was performed to assess the causal link between the consumption of AS from different origins and APOs. This research rigorously complied with the Strengthening the Reporting of OBservational studies in Epidemiology using Mendelian Randomization (STROBE-MR) guidelines and utilized the relevant checklist. Ethical approval and participant consent were secured from the initial studies. An overview of the flowchart can be found in Figure 1.

### 2.2. Data Source

Genome-wide association study (GWAS) summary data for exposure were obtained from the IEU Open GWAS Project [7] (v8.5.2, https://gwas.mrcieu.ac.uk/, accessed on 29 July 2024). Data on the intake of AS added to cereal, coffee, and tea were based on a GWAS involving 64,949 European participants. The evaluation of the phenotype was based on answers to questions such as “How much sweetener (e.g., Canderel) did you add to your cereal or porridge (per bowl)?” Participants’ responses included options of half a packet, 1 packet, 2 packets, 3+ packets, or varied. Data on BMI and T2DM, derived from self-reports, encompassed 336,107 and 462,933 European individuals, respectively. Data on fasting glucose were extracted from the Meta-Analysis of Glucose and Insulin-related traits Consortium (MAGIC), which included 133,010 European individuals [8]. Measures of fasting glucose taken from whole blood were adjusted to plasma levels using a correction factor of 1.13 [9]. GWAS summary data for APOs originated from the FinnGen consortium and the GWAS Catalog [10,11]. The outcome traits included ectopic pregnancy, excessive vomiting in pregnancy, gestational diabetes mellitus, intrahepatic cholestasis of pregnancy, medical abortion, pre-eclampsia, pregnancy hypertension, premature rupture of membranes (PROM), preterm birth, spontaneous abortion, placental disorders, placenta previa, abruptio placenta, pre-eclampsia or eclampsia, disorders related to short gestation and low birth weight, and disorders related to long gestation and high birth weight. The diagnostic criteria for these APOs were identified on the International Classification of Diseases, 10th Revision (ICD-10). All participants were of European descent, and the maximum sample overlap rate was 1.18%, with a type 1 error probability of less than 0.05 [12]. Detailed information for each dataset is presented in Table 1.

### 2.3. Selection of Instrumental Variables

The analysis of MR must adhere to three core assumptions: (1) the IV is strongly linked to the exposure; (2) the IV is not linked to any other potential confounders; and (3) the IV is not directly linked to the outcome, influencing it solely through the exposure. Consequently, the IVs selected from single-nucleotide polymorphisms (SNPs) satisfied the following criteria. (1) A statistically significant threshold of *p* < 5 × 10^−8^ was utilized for genome-wide significance to fulfill assumption 1. If no IVs remained, the *p*-value threshold was relaxed to 5 × 10^−6^. (2) IVs exhibiting linkage disequilibrium (LD) (r² > 0.001) were excluded using the clumping algorithm within a 10,000 kb window. (3) We computed F-statistic values for each IV, with the relevant equation presented below:

(1) $$F=\frac{{\mathrm{beta}}^{2}}{{\mathrm{se}}^{2}}$$

IVs with a Cragg–Donald F-statistic of less than 10 were excluded to mitigate weak instrumental variable bias [13]. (4) Palindromic IVs were excluded. (5) IVs associated with confounding factors and APOs were also excluded based on the LDtrait database (LDlink: An Interactive Web Tool for Exploring Linkage Disequilibrium in Population Groups). The confounding factors related to the exposures and outcomes included BMI, fasting glucose, T2DM, and intake of cereal, coffee, and tea.

In univariable MR (UVMR), we employed several methods, including inverse variance weighted (IVW), MR-Egger, weighted median, simple mode, and weighted mode, to evaluate the causal relationship between exposure and outcomes. IVW was the primary method for the statistical assessment of IVs and was regarded as the most powerful statistical analysis available [14]. MR-Egger identified causal relationships based on weak assumptions, particularly the Instrument Strength Independent of Direct Effect (InSIDE) assumption [15]. The weighted median technique necessitates that genetic factors account for a minimum of 50% of the overall weight, thus efficiently consolidating information from various genetic variables into a cohesive causal assessment. This method guarantees reliability in estimation, even if as much as 50% of the data come from unreliable IVs, and shows a better finite-sample type-I error rate than the IVW method [16]. The simple mode and weighted mode methods are limited to evaluating causal validity based solely on the cluster with the largest number of IVs, without the ability to estimate the bandwidth parameter [15]. A *p*-value threshold of 0.05 was established, with exposure considered a risk factor when the odds ratio (OR) exceeded 1 and a protective factor when the OR was less than 1. The heterogeneity of IVs selected in UVMR was assessed using the Cochran Q-statistic, applied through both the MR-Egger and IVW methods, with a *p*-value < 0.05 deemed statistically significant for the heterogeneity test. In cases where heterogeneity was detected among the IVs, a multiplicative random effects model of IVW (IVW-MRE) was implemented, and we further validated the results using the weighted median method to mitigate bias. Effect sizes for each MR method were visualized using scatter plots, while forest plots estimated the effect sizes for each IV and funnel plots illustrated the distributions of individual IV effects.

To further validate the consistency and robustness of the UVMR results, we conducted both multivariable MR (MVMR) and sensitivity analyses. MVMR is an extension of MR that utilizes genetic variants to assess the causal relationships between multiple related exposures and outcomes within the same model, thereby further mitigating potential confounding factors [17]. We performed MVMR to evaluate the impact of AS intake from various sources on APOs while adjusting for BMI and T2DM. Considering that all T2DM pregnant patients meet the diagnostic criteria of gestational diabetes, we changed the confounding factors as BMI and fasting glucose for adjustment when the outcome was gestational diabetes. The IVs used in the MVMR consisted of various combinations of IVs corresponding to each exposure, adhering to the selection criteria that were previously outlined, with the exception of (5). We applied the IVW, MR-Egger, and weighted median methods.

For UVMR, sensitivity analyses included the ‘leave-one-out’ method, pleiotropy test, and horizontal pleiotropy test. The ‘leave-one-out’ method was employed to assess the impact of each individual IV on the outcome by removing that IV and calculating the combined effect of the remaining IVs separately. Pleiotropy, defined as one locus influencing multiple phenotypes, can undermine the reliability of MR results. The MR-Egger intercept could analyze the pleiotropic effect of an IV [18]. The Mendelian Randomization Pleiotropy RESidual Sum and Outlier (MR-PRESSO) global test was applied to evaluate the horizontal pleiotropy. The MR-PRESSO outlier test specifically identified outliers for each IV, and outlier IVs were excluded if horizontal pleiotropy was detected. Subsequently, the MR analysis was conducted again. This approach ensured that MR-PRESSO provided corrected causal results free from the confounding effects of pleiotropy and outliers. For MVMR, MR-Egger intercept results were the assessment of pleiotropy. 

All statistical analyses were conducted with the help of the TwoSampleMR (version 0.5.8), MVMR (version 0.4), and ggpubr packages (version 0.6.0) utilized with R (version 4.3.1).

## 3. Results

### 3.1. Selection of IVs 

A rigorous screening process resulted in the selection of 41, 18, and 18 IVs associated with the intake of AS added to cereal, coffee, and tea, respectively. Detailed information regarding each IV is available in Table S1.

### 3.2. UVMR

**Figure 2:**
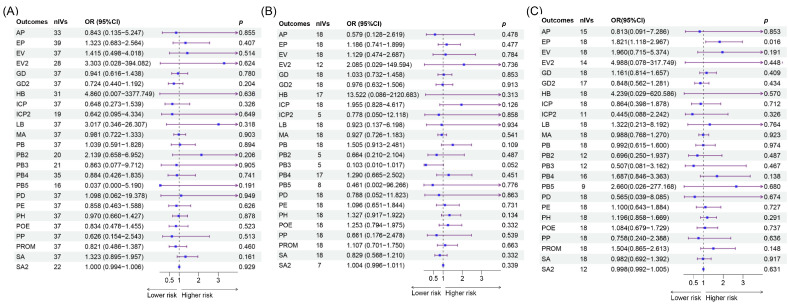
Causal relationships between the intake of AS and APOs in UVMR. (**A**) Results of IVW between the intake of AS added to cereal and APOs. (**B**) Results of IVW between the intake of AS added to coffee and APOs. (**C**) Results of IVW between the intake of AS added to tea and APOs. AS, artificial sweetener; APOs, adverse pregnancy outcomes; UVMR, univariable Mendelian randomization; IVW, inverse variance weighted; AP, abruptio placenta (O15_PLAC_PREMAT_SEPAR); EP, ectopic pregnancy (GCST90272883); EV, excessive vomiting in pregnancy (O15_EXCESS_VOMIT_PREG); EV2, excessive vomiting in pregnancy (GCST90044480); GDM, gestational diabetes mellitus (GEST_DIABETES); GDM2, gestational diabetes mellitus (GCST90296696); HB, disorders associated with long gestation and high birth weight (R10_P16_DISORD_RELATED_LONG_GESTATION_HIGH_BIRTHWGHTT); ICP2, intrahepatic cholestasis of pregnancy (O15_ICP_WIDE); ICP2, intrahepatic cholestasis of pregnancy (GCST90095084); LB, disorders related to short gestation and low birth weight (R10_P16_DISORD_RELATED_GESTATION_LOW_BIRTHWGHTT_NECIFIED); MD, medical abortion (O15_ABORT_MEDICAL); PB, preterm birth (O15_PRETERM); PB2, preterm birth (GCST008754); PB3, preterm birth (GCST008753); PB4, preterm birth (GCST90271753); PB5, preterm birth (GCST90271755); PD, placental disorders (O15_PLAC_DISORD); PE, pre-eclampsia (O15_PREECLAMPS); PH, pregnancy hypertension (O15_HYPTENSPREG); POE, pre-eclampsia or eclampsia (O15_PRE_OR_ECLAMPSIA); PP, placenta previa (O15_PLAC_PRAEVIA); PROM, premature rupture of membranes (O15_MEMBR_PREMAT_RUPT); SA, spontaneous abortion (O15_ABORT_SPONTAN); SA2, spontaneous abortion (ukb-d-O03).

A causal effect of the intake of AS added to tea on ectopic pregnancy was observed [OR = 1.821(1.118–2.967), *p* = 0.016]. No causal relationships were identified between the intake of AS from different sources and the other APOs (Figure 2, Figures S1–S3). The IVW-MRE method was employed, and the weighted median results were verified when heterogeneity was detected, yielding consistent outcomes (Tables S2 and S3). The robustness of our findings was further confirmed by forest plots, funnel plots, and the leave-one-out method (Figures S4–S12). Pleiotropy was observed when the exposure was the intake of AS added from tea and the outcomes were placenta previa and spontaneous abortion, as indicated by the MR-Egger intercept or the MR-PRESSO global test (Table S4). However, outlier analysis did not identify any outlier instrumental variables.

### 3.3. MVMR

**Figure 3:**
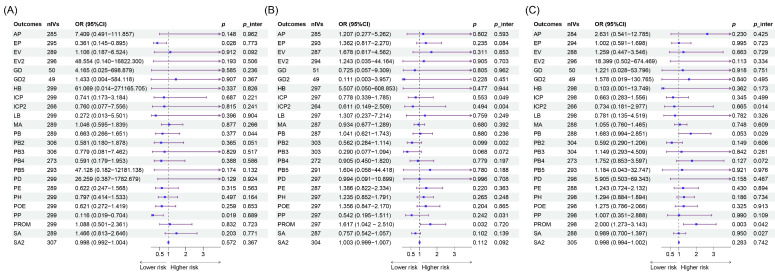
Causal relationships between the intake of AS adjusting for confounders and APOs in MVMR. (**A**) Results of IVW between the intake of AS added to cereal and APOs. (**B**) Results of IVW between the intake of AS added to coffee and APOs. (**C**) Results of IVW between the intake of AS added to tea and APOs. AS, artificial sweetener; APOs, adverse pregnancy outcomes; MVMR, multivariable Mendelian randomization; IVW, inverse variance weighted; AP, abruptio placenta (O15_PLAC_PREMAT_SEPAR); EP, ectopic pregnancy (GCST90272883); EV, excessive vomiting in pregnancy (O15_EXCESS_VOMIT_PREG); EV2, excessive vomiting in pregnancy (GCST90044480); GDM, gestational diabetes mellitus (GEST_DIABETES); GDM2, gestational diabetes mellitus (GCST90296696); HB, disorders associated with long gestation and high birth weight (R10_P16_DISORD_RELATED_LONG_GESTATION_HIGH_BIRTHWGHTT); ICP2, intrahepatic cholestasis of pregnancy (O15_ICP_WIDE); ICP2, intrahepatic cholestasis of pregnancy (GCST90095084); LB, disorders related to short gestation and low birth weight (R10_P16_DISORD_RELATED_GESTATION_LOW_BIRTHWGHTT_NECIFIED); MD, medical abortion (O15_ABORT_MEDICAL); PB, preterm birth (O15_PRETERM); PB2, preterm birth (GCST008754); PB3, preterm birth (GCST008753); PB4, preterm birth (GCST90271753); PB5, preterm birth (GCST90271755); PD, placental disorders (O15_PLAC_DISORD); PE, pre-eclampsia (O15_PREECLAMPS); PH, pregnancy hypertension (O15_HYPTENSPREG); POE, pre-eclampsia or eclampsia (O15_PRE_OR_ECLAMPSIA); PP, placenta previa (O15_PLAC_PRAEVIA); PROM, premature rupture of membranes (O15_MEMBR_PREMAT_RUPT); SA, spontaneous abortion (O15_ABORT_SPONTAN); SA2, spontaneous abortion (ukb-d-O03).

After adjusting for BMI and T2DM, causal effects were detected in the intake of AS added to cereal on ectopic pregnancy [OR = 0.361(0.145–0.895), *p* = 0.028] and placenta previa [OR = 0.116(0.019–0.704), *p* = 0.019] (Figure 3, Tables S5 and S6). Additionally, significant effects were noted for the intake of AS added to coffee on PROM [OR = 1.617 (1.042–2.510), *p* = 0.032]. The results from the MR-Egger intercept indicated pleiotropy in some analyses (Figure 3).

**Figure 4:**
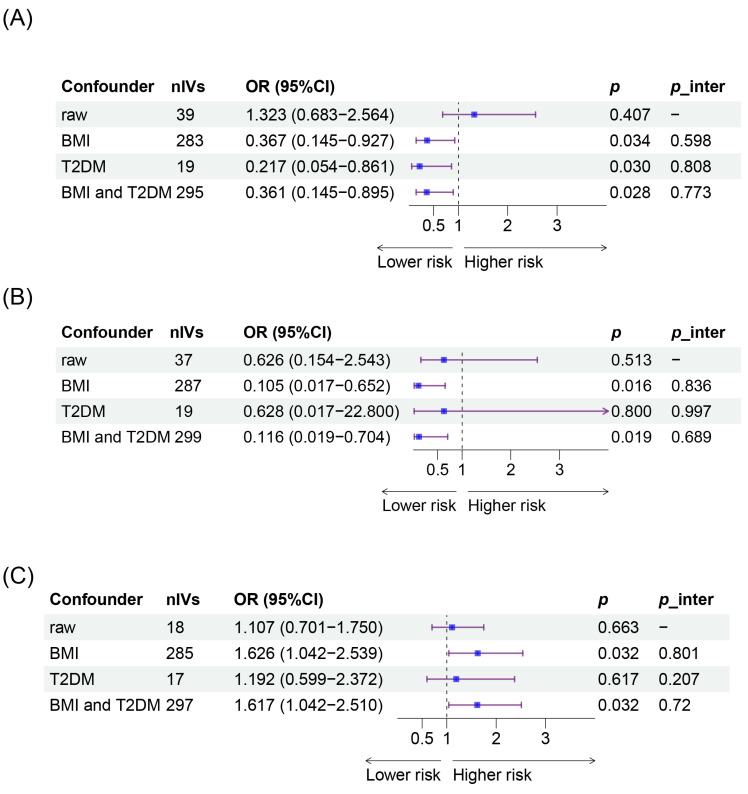
Causal relationships between the intake of AS and APOs in UVMR and MVMR. (**A**) Results of IVW between the intake of AS added to cereal and ectopic pregnancy adjusting for different confounders. (**B**) Results of IVW between the intake of AS added to cereal and placenta previa adjusting for different confounders. (**C**) Results of IVW between the intake of AS added to coffee and PROM adjusting for different confounders. AS, artificial sweetener; APOs, adverse pregnancy outcomes; UVMR, univariable Mendelian randomization; MVMR, multivariable Mendelian randomization; IVW, inverse variance weighted; PROM, premature rupture of membranes.

We then conducted further MVMR by adjusting for BMI and T2DM in the other exposures and outcomes where the causal effects were significant (Figure 4, Table S7). The adjustments for BMI yielded results consistent with the aforementioned findings. The causal effect of the intake of AS added to cereal on ectopic pregnancy remained consistent when adjusting for T2DM.

## 4. Discussion

Our study is the first to systematically analyze the causal relationship between the intake of AS from various sources and APOs at the genetic variation level. The results of UVMR suggest that the intake of AS added to tea may increase the risk of ectopic pregnancy. The MVMR results indicate that the intake of AS added to cereal may also reduce the risk of ectopic pregnancy and placenta previa. Furthermore, the intake of AS added to coffee may elevate the risk of PROM.

In the Randomized Control Trial of Low Glycemic Index Diet in Pregnancy to Prevent Recurrence of Macrosomia (ROLO), one-third of pregnant women reported consuming AS during each trimester [19]. This proportion increased to 51.4% after receiving advice on a low glycemic index diet, suggesting that AS intake is quite prevalent during pregnancy. Studies from various regions have shown that the AS intake among pregnant women is consistent with, or even exceeds, that of the general population [6,20,21,22,23]. Therefore, the potential impact of AS intake on APOs represents a significant research question.

Current research on the relationship between AS and APOs in pregnant women is limited and inconsistent. Studies conducted in animal models have demonstrated that aspartame consumption during pregnancy may lead to impaired glucose tolerance, weight gain, and cognitive impairments [24,25]. However, a meta-analysis encompassing 24 studies found that most did not identify any significant impact of AS [26]. In human studies, the consumption of diet drinks during pregnancy has been linked to increased risks of gestational weight gain, gestational diabetes, and pre-eclampsia [3,27,28]. The relationship between AS and preterm birth remains controversial [4,29]. Most of these studies are observational, making it challenging to establish causality. Prospective cohort studies frequently rely on Food Frequency Questionnaires (FFQs) instead of more rigorous 24 h dietary recalls or dietary provisions, which can introduce recall bias. The limitations inherent in study design hinder the establishment of causality and the exclusion of confounding factors, resulting in inconsistent findings. To address these issues, we conducted a two-sample MR analysis utilizing GWAS data to minimize confounding and reverse causation. Recognizing that AS intake and APOs may be related to BMI and glucose metabolism, we also performed MVMR to elucidate their independent causal effects. Furthermore, our study included sensitivity analyses to enhance the stability and reliability of the results. Ultimately, our findings do not support the causal effects of AS intake on the aforementioned outcomes.

Ectopic pregnancy and placenta previa are associated with maternal morbidity. Currently, there are no epidemiological studies examining the relationship between AS intake and these outcomes. In UVMR analysis, the intake of AS added to tea was found to increase the risk of ectopic pregnancy; however, this association lacked consistency in MVMR results. When adjusting for BMI and T2DM, the intake of AS added to cereal significantly reduced the incidence of both ectopic pregnancy and placenta previa. The results remained stable when adjusting for BMI, and the causal effects remained consistent in ectopic pregnancy when adjusting for T2DM. Nevertheless, no significant effects on these outcomes were observed in the UVMR analysis, potentially because of a preference for AS intake added to cereal among individuals with higher BMI or T2DM. Pregnant women with higher BMI or T2DM may utilize AS to decrease overall sugar intake during pregnancy, which could aid in controlling blood sugar levels and weight. Concurrently, studies have indicated that the consumption of artificial sweetener does not significantly affect appetite [30,31], suggesting that this mechanism may contribute to a reduced risk of ectopic pregnancy and placenta previa.

PROM is one of the most common causes of preterm birth. Our study found that, after adjusting for BMI and T2DM, the intake of AS added to coffee is a risk factor for PROM. Currently, there are no epidemiological studies investigating the relationship between AS intake and PROM. Previous cross-sectional studies have indicated that coffee consumption during pregnancy is associated with PROM. Women who are pregnant and drink three or more cups of coffee each day in the first trimester face more than twice the risk of PROM in comparison with those who have fewer than three cups [32]. Therefore, the impact of AS added to coffee on PROM may be attributed to an increase in overall coffee consumption among pregnant women.

Our study has several limitations. (1) Our research exclusively included individuals of European ancestry, which may result in disease incidence and dietary preferences that are specific to this group. This limitation affects the generalizability of our conclusions to other populations. Nonetheless, we incorporated as much outcome data as possible to enhance the reliability of our findings and their applicability to various European populations. (2) Because of the constraints of GWAS summary-level data, we were unable to conduct subgroup analyses to investigate potential stratified effects of factors such as age, health status, glucose levels, and insulin resistance. (3) We focused solely on BMI, fasting glucose, and T2DM as confounding factors; however, other potential confounders that may affect the relationship between AS intake and APOs warrant further exploration. Future studies should aim to expand the study population to validate the potential impact of AS intake on placenta previa and PROM. Additionally, further research is necessary to investigate the underlying mechanisms, which will provide guidance on AS intake for women of reproductive age.

## 5. Conclusions

Our study identified causal effects of AS intake on placenta previa and PROM. Further randomized clinical trials are needed to confirm this potential causality.

## Figures and Tables

**Table 1:** The list of genome-wide association studies included in the Mendelian randomization study.

Traits	Consortium	Sample Size (Cases/Controls)	Population	GWAS ID and Web Link (Accessed on 29 July 2024)
Exposure				
Intake of artificial sweetener added to cereal	MRC-IEU	64,949	European	ukb-b-3143 (https://gwas.mrcieu.ac.uk/datasets/ukb-b-3143/)
Intake of artificial sweetener added to coffee	MRC-IEU	64,949		ukb-b-1338 (https://gwas.mrcieu.ac.uk/datasets/ukb-b-1338/)
Intake of artificial sweetener added to tea	MRC-IEU	64,949		ukb-b-5867 (https://gwas.mrcieu.ac.uk/datasets/ukb-b-5867/)
Body mass index	Neale Lab	336,107		ukb-a-248 (https://gwas.mrcieu.ac.uk/datasets/ukb-a-248/)
Type 2 diabetes	MRC-IEU	462,933		ukb-b-13806 (https://gwas.mrcieu.ac.uk/datasets/ukb-b-13806/)
Fasting glucose	MAGIC	133,010		ieu-b-114 (https://gwas.mrcieu.ac.uk/datasets/ieu-b-114/)
Outcome				
Ectopic pregnancy	GWAS Catalog	7070/248,810	European	GCST90272883 (https://ftp.ebi.ac.uk/pub/databases/gwas/summary_statistics/GCST90272001-GCST90273000/GCST90272883/)
Excessive vomiting in pregnancy	FinnGen	2092/163,702		O15_EXCESS_VOMIT_PREG (https://storage.googleapis.com/finngen-public-data-r10/summary_stats/finngen_R10_O15_EXCESS_VOMIT_PREG.gz)
	GWAS Catalog	146/247,394		GCST90044480 (https://ftp.ebi.ac.uk/pub/databases/gwas/summary_statistics/GCST90044001-GCST90045000/GCST90044480/)
Gestational diabetes mellitus	FinnGen	11,279/179,600		GEST_DIABETES (https://storage.googleapis.com/finngen-public-data-r10/summary_stats/finngen_R10_GEST_DIABETES.gz)
	GWAS Catalog	12,332/131,109		GCST90296696 (https://ftp.ebi.ac.uk/pub/databases/gwas/summary_statistics/GCST90296001-GCST90297000/GCST90296696/)
Intrahepatic cholestasis of pregnancy	FinnGen	2196/188,683		O15_ICP_WIDE (https://storage.googleapis.com/finngen-public-data-r10/summary_stats/finngen_R10_O15_ICP_WIDE.gz)
	GWAS Catalog	1138/153,642		GCST90095084 (https://ftp.ebi.ac.uk/pub/databases/gwas/summary_statistics/GCST90095001-GCST90096000/GCST90095084/)
Medical abortion	FinnGen	32,550/135,962		O15_ABORT_MEDICAL (https://storage.googleapis.com/finngen-public-data-r10/summary_stats/finngen_R10_O15_ABORT_MEDICAL.gz)
Preeclampsia	FinnGen	5922/176,113		O15_PREECLAMPS (https://storage.googleapis.com/finngen-public-data-r10/summary_stats/finngen_R10_O15_PREECLAMPS.gz)
Pregnancy hypertension	FinnGen	13,071/177,808		O15_HYPTENSPREG (https://storage.googleapis.com/finngen-public-data-r10/summary_stats/finngen_R10_O15_HYPTENSPREG.gz)
Premature rupture of membranes	FinnGen	6129/154,102		O15_MEMBR_PREMAT_RUPT (https://storage.googleapis.com/finngen-public-data-r10/summary_stats/finngen_R10_O15_MEMBR_PREMAT_RUPT.gz)
Preterm birth	FinnGen	7678/148,153		O15_PRETERM (https://storage.googleapis.com/finngen-public-data-r10/summary_stats/finngen_R10_O15_PRETERM.gz)
	GWAS Catalog	4775/60,148		GCST008754 (https://ftp.ebi.ac.uk/pub/databases/gwas/summary_statistics/GCST008001-GCST009000/GCST008754/)
	GWAS Catalog	1139/60,148		GCST008753 (https://ftp.ebi.ac.uk/pub/databases/gwas/summary_statistics/GCST008001-GCST009000/GCST008753/)
	GWAS Catalog	4925/49,105		GCST90271753 (https://ftp.ebi.ac.uk/pub/databases/gwas/summary_statistics/GCST90271001-GCST90272000/GCST90271753/)
	GWAS Catalog	286/488		GCST90271755 (https://ftp.ebi.ac.uk/pub/databases/gwas/summary_statistics/GCST90271001-GCST90272000/GCST90271755/)
Spontaneous abortion	FinnGen	15,073/135,962		O15_ABORT_SPONTAN (https://storage.googleapis.com/finngen-public-data-r10/summary_stats/finngen_R10_O15_ABORT_SPONTAN.gz)
	Neale Lab	1150/360,044		ukb-d-O03 (https://gwas.mrcieu.ac.uk/datasets/ukb-d-O03/)
Placental disorders	FinnGen	253/182,824		O15_PLAC_DISORD (https://storage.googleapis.com/finngen-public-data-r10/summary_stats/finngen_R10_O15_PLAC_DISORD.gz)
Placenta previa	FinnGen	1400/182,824		O15_PLAC_PRAEVIA (https://storage.googleapis.com/finngen-public-data-r10/summary_stats/finngen_R10_O15_PLAC_PRAEVIA.gz)
Abruptio placenta	FinnGen	691/182,824		O15_PLAC_PREMAT_SEPAR (https://storage.googleapis.com/finngen-public-data-r10/summary_stats/finngen_R10_O15_PLAC_PREMAT_SEPAR.gz)
Preeclampsia or eclampsia	FinnGen	7965/211,852		O15_PRE_OR_ECLAMPSIA (https://storage.googleapis.com/finngen-public-data-r10/summary_stats/finngen_R10_O15_PRE_OR_ECLAMPSIA.gz)
Disorders related to short gestation and low birth weight	FinnGen	573/411,504		R10_P16_DISORD_RELATED_GESTATION_LOW_BIRTHWGHTT_NECIFIED (https://storage.googleapis.com/finngen-public-data-r10/summary_stats/finngen_R10_P16_DISORD_RELATED_GESTATION_LOW_BIRTHWGHTT_NECIFIED.gz)
Disorders related to long gestation and high birth weight	FinnGen	65/411,504		R10_P16_DISORD_RELATED_LONG_GESTATION_HIGH_BIRTHWGHTT (https://storage.googleapis.com/finngen-public-data-r10/summary_stats/finngen_R10_P16_DISORD_RELATED_LONG_GESTATION_HIGH_BIRTHWGHTT.gz)

MRC-IEU, Medical Research Council Integrative Epidemiology Unit at the University of Bristol; MAGIC, Meta-Analyses of Glucose and Insulin-related traits Consortium.

## Data Availability

The original data presented in the study are openly available in the IEU Open GWAS repository [7] (v8.5.2, https://gwas.mrcieu.ac.uk/, accessed on 29 July 2024), FinnGen repository [10] (https://www.finngen.fi/en/access_results, accessed on 29 July 2024) and GWAS Catalog repository [11] (https://www.ebi.ac.uk/gwas/downloads/summary-statistics, accessed on 29 July 2024).

## Supplementary Materials

The following supporting information can be downloaded at: https://www.mdpi.com/article/10.3390/nu16193366/s1, Figure S1. Scatter plots of univariable Mendelian randomization when exposure is the intake of artificial sweetener added to cereal. Figure S2. Scatter plots of univariable Mendelian randomization when exposure is intake of artificial sweetener added to coffee. Figure S3. Scatter plots of univariable Mendelian randomization when exposure is intake of artificial sweetener added to tea. Figure S4. Forest plots for each single-nucleotide polymorphism of univariable Mendelian randomization when exposure is intake of artificial sweetener added to cereal. Figure S5. Forest plots for each single-nucleotide polymorphism of univariable Mendelian randomization when exposure is intake of artificial sweetener added to coffee. Figure S6. Forest plots for each single-nucleotide polymorphism of univariable Mendelian randomization when exposure is intake of artificial sweetener added to cereal. Figure S7. Funnel plots for of univariable Mendelian randomization when exposure is intake of artificial sweetener added to cereal. Figure S8. Funnel plots for of univariable Mendelian randomization when exposure is intake of artificial sweetener added to coffee. Figure S9. Funnel plots for of univariable Mendelian randomization when exposure is intake of artificial sweetener added to tea. Figure S10. Results of ‘leave-one-out’ of univariable Mendelian randomization when exposure is intake of artificial sweetener added to cereal. Figure S11. Results of ‘leave-one-out’ of univariable Mendelian randomization when exposure is intake of artificial sweetener added to coffee. Figure S12. Results of ‘leave-one-out’ of univariable Mendelian randomization when exposure is intake of artificial sweetener added to tea. Table S1. Information on instrumental variables selected in univariable Mendelian randomization. Table S2. Results of univariable Mendelian randomization in various methods. Table S3. Results of pleiority analysis in univariable Mendelian randomization. Table S4. Results of sensitivity analysis in univariable Mendelian randomization. Table S5. Information on instrumental variables selected in body mass index, fasting glucose, and type 2 diabetes mellitus. Table S6. Results of multivariable Mendelian randomization in various methods. Table S7. Results of multivariable Mendelian randomization adjusting for body mass index or type 2 diabetes mellitus in various methods.
