# Supplementary material for: Artificial Sweetener and the Risk of Adverse Pregnancy Outcomes: A Mendelian Randomization Study
Source: Nutrients. 2024 Oct 3;16(19):3366. doi: 10.3390/nu16193366 (PMC11479087; doi:10.3390/nu16193366)
Supplement: Supplementary file 1 [file nutrients-16-03366-s001.zip › Supplement 1.pdf]

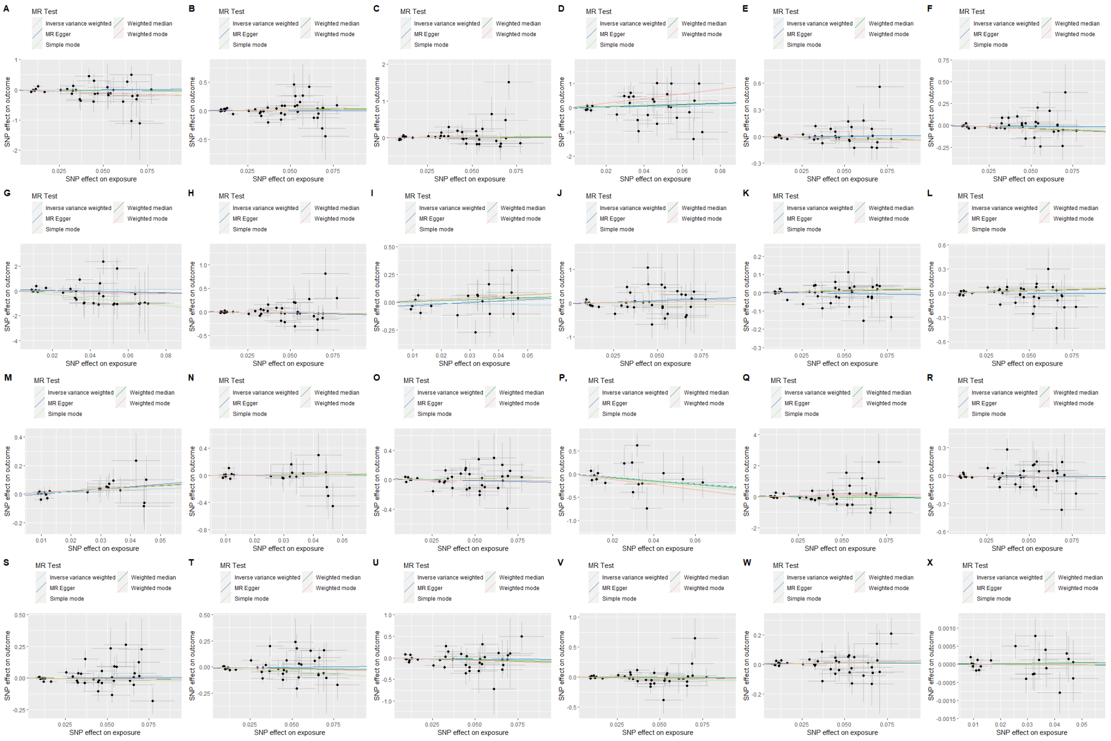

Figure S1. The scatter plots of univariable mendelian randomization when exposure is the intake of artificial sweetener added to cereal. (A)-(X) refers the scatter plot of each APOs including abruption placenta (O15_PLAC_PREMAT_SEPAR), ectopic pregnancy (GCST90272883), excessive vomiting in pregnancy (O15_EXCESS_VOMIT_PREG), excessive vomiting in pregnancy (GCST90044480), gestational diabetes mellitus (GEST_DIABETES), gestational diabetes mellitus (GCST90296696), disorders associated with long gestation and high birth weight (R10_P16_DISORD_RELATED_LONG_GESTATION_HIGH_BIRTHWGHTT), intrahepatic cholestasis of pregnancy (O15_ICP_WIDE), intrahepatic cholestasis of pregnancy (GCST90095084), disorders related to short gestation and low birth weight (R10_P16_DISORD_RELATED_GESTATION_LOW_BIRTHWGHTT_NECIFIED), medical abortion (O15_ABORT_MEDICAL), preterm birth (O15_PRETERM), preterm birth (GCST008754), preterm birth (GCST008753), preterm birth (GCST90271753), preterm birth (GCST90271755), placental disorders (O15_PLAC_DISORD), preeclampsia (O15_PREECLAMPS), pregnancy hypertension (O15_HYPTENSPREG), pre-eclampsia or eclampsia (O15_PRE_OR_ECLAMPSIA), placenta previa (O15_PLAC_PREVIA), premature rupture of membranes (O15_MEMBR_PREMAT_RUPT), spontaneous abortion (O15_ABORT_SPONTAN) and spontaneous abortion (ukb-d-O03) respectively. APOs, adverse pregnancy outcomes.

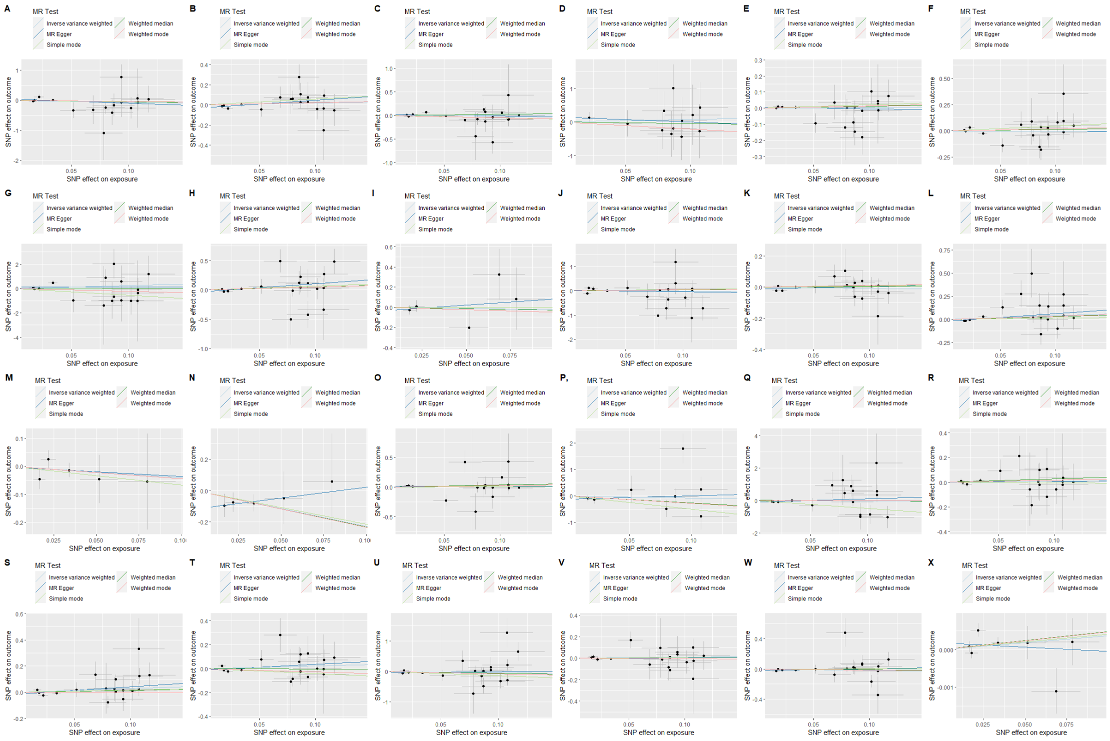

Figure S2. The scatter plots of univariable mendelian randomization when exposure is the intake of artificial sweetener added to coffee. (A)-(X) refers the scatter plot of each APOs including abruption placenta (O15_PLAC_PREMAT_SEPAR), ectopic pregnancy (GCST90272883), excessive vomiting in pregnancy (O15_EXCESS_VOMIT_PREG), excessive vomiting in pregnancy (GCST90044480), gestational diabetes mellitus (GEST_DIABETES), gestational diabetes mellitus (GCST90296696), disorders associated with long gestation and high birth weight (R10_P16_DISORD_RELATED_LONG_GESTATION_HIGH_BIRTHWGHTT), intrahepatic cholestasis of pregnancy (O15_ICP_WIDE), intrahepatic cholestasis of pregnancy (GCST90095084), disorders related to short gestation and low birth weight (R10_P16_DISORD_RELATED_GESTATION_LOW_BIRTHWGHTT_NECIFIED), medical abortion (O15_ABORT_MEDICAL), preterm birth (O15_PRETERM), preterm birth (GCST008754), preterm birth (GCST008753), preterm birth (GCST90271753), preterm birth (GCST90271755), placental disorders (O15_PLAC_DISORD), preeclampsia (O15_PREECLAMPS), pregnancy hypertension (O15_HYPTENSPREG), pre-eclampsia or eclampsia (O15_PRE_OR_ECLAMPSIA), placenta previa (O15_PLAC_PREVIA), premature rupture of membranes (O15_MEMBR_PREMAT_RUPT), spontaneous abortion (O15_ABORT_SPONTAN) and spontaneous abortion (ukb-d-O03) respectively. APOs, adverse pregnancy outcomes.

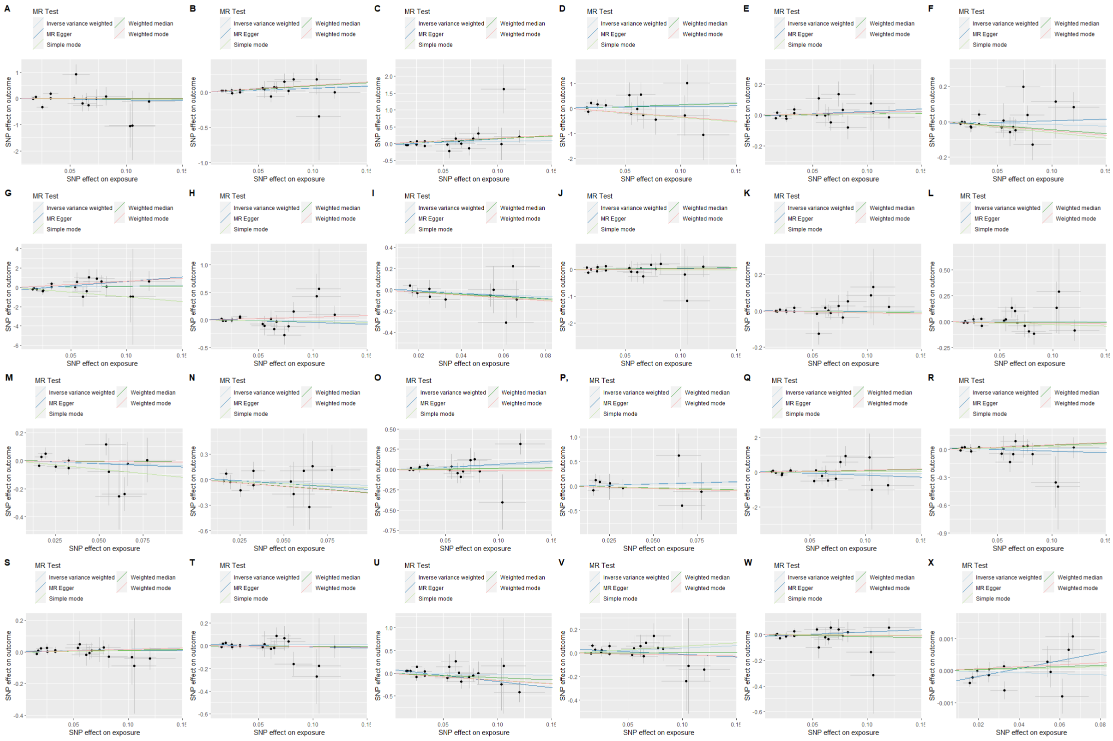

Figure S3. The scatter plots of univariable mendelian randomization when exposure is the intake of artificial sweetener added to tea. (A)-(X) refers the scatter plot of each APOs including abruption placenta (O15_PLAC_PREMAT_SEPAR), ectopic pregnancy (GCST90272883), excessive vomiting in pregnancy (O15_EXCESS_VOMIT_PREG), excessive vomiting in pregnancy (GCST90044480), gestational diabetes mellitus (GEST_DIABETES), gestational diabetes mellitus (GCST90296696), disorders associated with long gestation and high birth weight (R10_P16_DISORD_RELATED_LONG_GESTATION_HIGH_BIRTHWGHTT), intrahepatic cholestasis of pregnancy (O15_ICP_WIDE), intrahepatic cholestasis of pregnancy (GCST90095084), disorders related to short gestation and low birth weight (R10_P16_DISORD_RELATED_GESTATION_LOW_BIRTHWGHTT_NECIFIED), medical abortion (O15_ABORT_MEDICAL), preterm birth (O15_PRETERM), preterm birth (GCST008754), preterm birth (GCST008753), preterm birth (GCST90271753), preterm birth (GCST90271755), placental disorders (O15_PLAC_DISORD), preeclampsia (O15_PREECLAMPS), pregnancy hypertension (O15_HYPTENSPREG), pre-eclampsia or eclampsia (O15_PRE_OR_ECLAMPSIA), placenta previa (O15_PLAC_PREVIA), premature rupture of membranes (O15_MEMBR_PREMAT_RUPT), spontaneous abortion (O15_ABORT_SPONTAN) and spontaneous abortion (ukb-d-O03) respectively. APOs, adverse pregnancy outcomes.

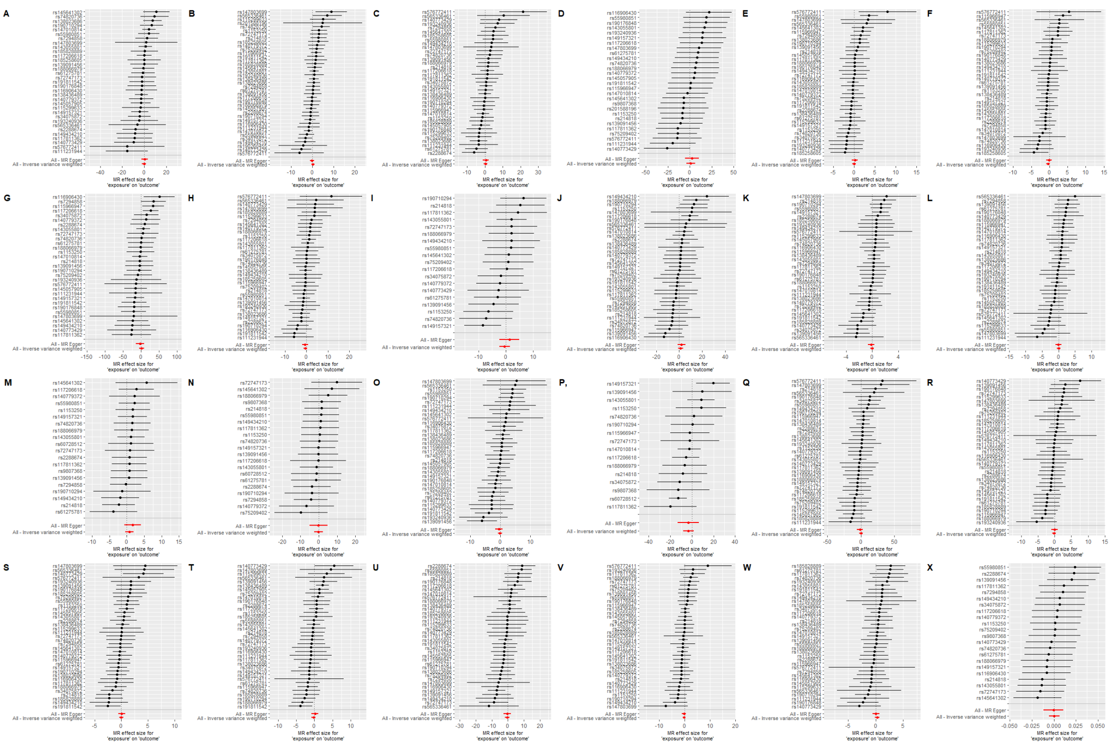

Figure S4. The forest plots for each single nucleotide polymorphism of univariable mendelian randomization when exposure is the intake of artificial sweetener added to cereal. (A)-(X) refers the forest plot of each APOs including abruption placenta (O15_PLAC_PREMAT_SEPAR), ectopic pregnancy (GCST90272883), excessive vomiting in pregnancy (O15_EXCESS_VOMIT_PREG), excessive vomiting in pregnancy (GCST90044480), gestational diabetes mellitus (GEST_DIABETES), gestational diabetes mellitus (GCST90296696), disorders associated with long gestation and high birth weight (R10_P16_DISORD_RELATED_LONG_GESTATION_HIGH_BIRTHWGHTT), intrahepatic cholestasis of pregnancy (O15_ICP_WIDE), intrahepatic cholestasis of pregnancy (GCST90095084), disorders related to short gestation and low birth weight (R10_P16_DISORD_RELATED_GESTATION_LOW_BIRTHWGHTT_NECIFIED), medical abortion (O15_ABORT_MEDICAL), preterm birth (O15_PRETERM), preterm birth (GCST008754), preterm birth (GCST008753), preterm birth (GCST90271753), preterm birth (GCST90271755), placental disorders (O15_PLAC_DISORD), preeclampsia (O15_PREECLAMPS), pregnancy hypertension (O15_HYPTENSPREG), pre-eclampsia or eclampsia (O15_PRE_OR_ECLAMPSIA), placenta previa (O15_PLAC_PREVIA), premature rupture of membranes (O15_MEMBR_PREMAT_RUPT), spontaneous abortion (O15_ABORT_SPONTAN) and spontaneous abortion (ukb-d-O03) respectively. APOs, adverse pregnancy outcomes.

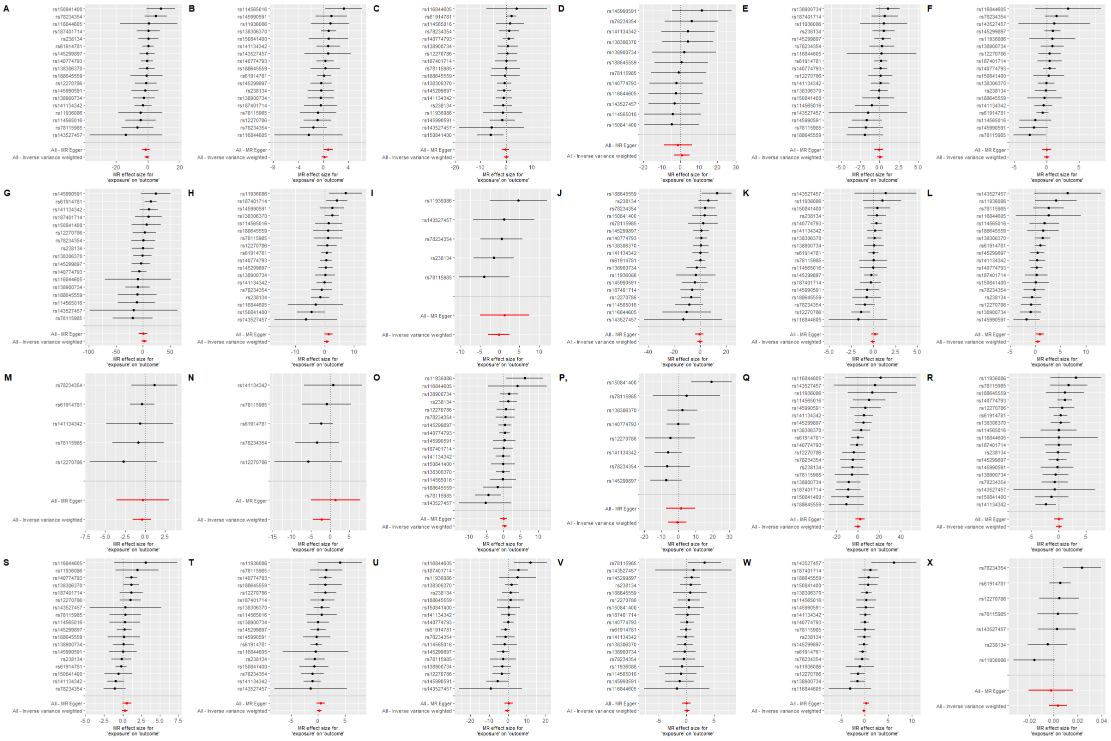

Figure S5. The forest plots for each single nucleotide polymorphism of univariable mendelian randomization when exposure is the intake of artificial sweetener added to coffee. (A)-(X) refers the forest plot of each APOs including abruption placenta (O15_PLAC_PREMAT_SEPAR), ectopic pregnancy (GCST90272883), excessive vomiting in pregnancy (O15_EXCESS_VOMIT_PREG), excessive vomiting in pregnancy (GCST90044480), gestational diabetes mellitus (GEST_DIABETES), gestational diabetes mellitus (GCST90296696), disorders associated with long gestation and high birth weight (R10_P16_DISORD_RELATED_LONG_GESTATION_HIGH_BIRTHWGHTT), intrahepatic cholestasis of pregnancy (O15_ICP_WIDE), intrahepatic cholestasis of pregnancy (GCST90095084), disorders related to short gestation and low birth weight (R10_P16_DISORD_RELATED_GESTATION_LOW_BIRTHWGHTT_NECIFIED), medical abortion (O15_ABORT_MEDICAL), preterm birth (O15_PRETERM), preterm birth (GCST008754), preterm birth (GCST008753), preterm birth (GCST90271753), preterm birth (GCST90271755), placental disorders (O15_PLAC_DISORD), preeclampsia (O15_PREECLAMPS), pregnancy hypertension (O15_HYPTENSPREG), pre-eclampsia or eclampsia (O15_PRE_OR_ECLAMPSIA), placenta previa (O15_PLAC_PREVIA), premature rupture of membranes (O15_MEMBR_PREMAT_RUPT), spontaneous abortion (O15_ABORT_SPONTAN) and spontaneous abortion (ukb-d-O03) respectively. APOs, adverse pregnancy outcomes.

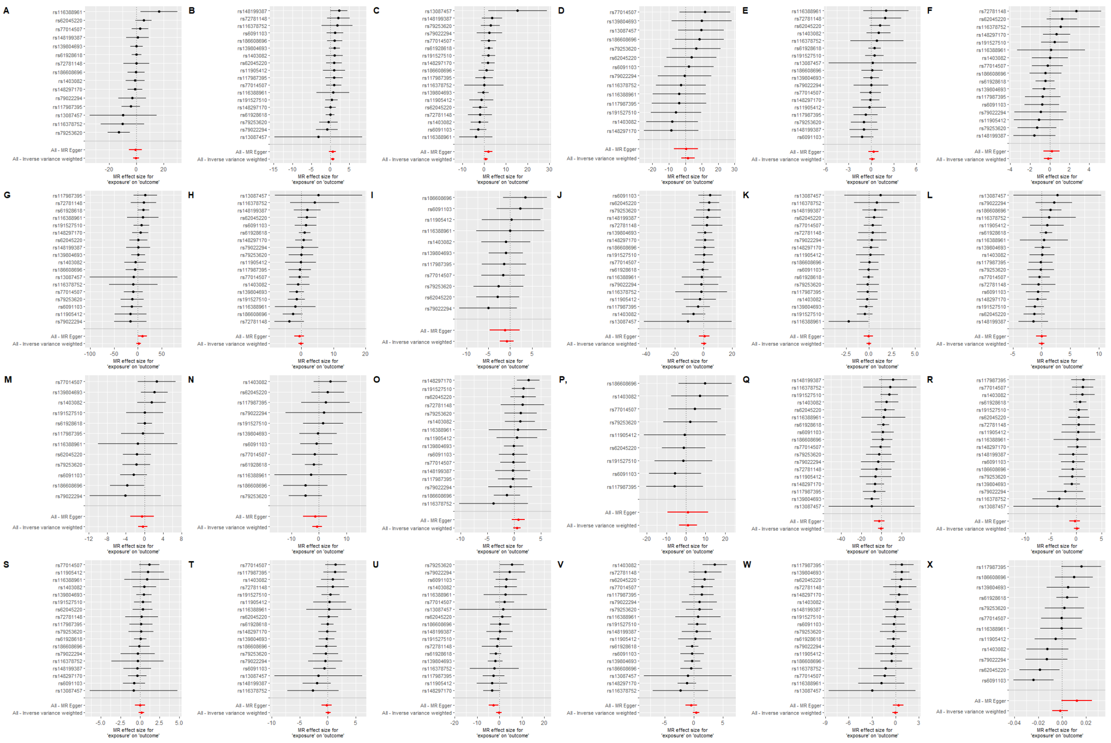

Figure S6. The forest plots for each single nucleotide polymorphism of univariable mendelian randomization when exposure is the intake of artificial sweetener added to tea. (A)-(X) refers the forest plot of each APOs including abruption placenta (O15_PLAC_PREMAT_SEPAR), ectopic pregnancy (GCST90272883), excessive vomiting in pregnancy (O15_EXCESS_VOMIT_PREG), excessive vomiting in pregnancy (GCST90044480), gestational diabetes mellitus (GEST_DIABETES), gestational diabetes mellitus (GCST90296696), disorders associated with long gestation and high birth weight (R10_P16_DISORD_RELATED_LONG_GESTATION_HIGH_BIRTHWGHTT), intrahepatic cholestasis of pregnancy (O15_ICP_WIDE), intrahepatic cholestasis of pregnancy (GCST90095084), disorders related to short gestation and low birth weight (R10_P16_DISORD_RELATED_GESTATION_LOW_BIRTHWGHTT_NECIFIED), medical abortion (O15_ABORT_MEDICAL), preterm birth (O15_PRETERM), preterm birth (GCST008754), preterm birth (GCST008753), preterm birth (GCST90271753), preterm birth (GCST90271755), placental disorders (O15_PLAC_DISORD), preeclampsia (O15_PREECLAMPS), pregnancy hypertension (O15_HYPTENSPREG), pre-eclampsia or eclampsia (O15_PRE_OR_ECLAMPSIA), placenta previa (O15_PLAC_PREVIA), premature rupture of membranes (O15_MEMBR_PREMAT_RUPT), spontaneous abortion (O15_ABORT_SPONTAN) and spontaneous abortion (ukb-d-O03) respectively. APOs, adverse pregnancy outcomes.

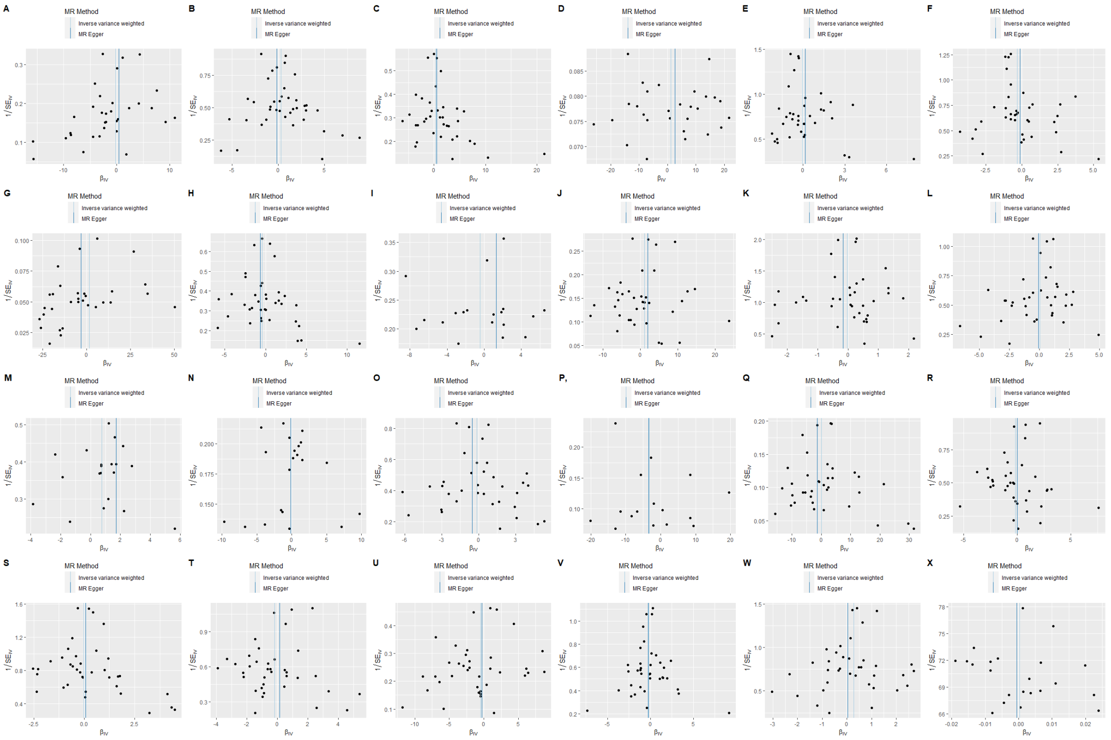

Figure S7. The funnel plots for of univariable mendelian randomization when exposure is the intake of artificial sweetener added to cereal. (A)-(X) refers the funnel plot of each APOs including abruption placenta (O15_PLAC_PREMAT_SEPAR), ectopic pregnancy (GCST90272883), excessive vomiting in pregnancy (O15_EXCESS_VOMIT_PREG), excessive vomiting in pregnancy (GCST90044480), gestational diabetes mellitus (GEST_DIABETES), gestational diabetes mellitus (GCST90296696), disorders associated with long gestation and high birth weight (R10_P16_DISORD_RELATED_LONG_GESTATION_HIGH_BIRTHWGHTT), intrahepatic cholestasis of pregnancy (O15_ICP_WIDE), intrahepatic cholestasis of pregnancy (GCST90095084), disorders related to short gestation and low birth weight (R10_P16_DISORD_RELATED_GESTATION_LOW_BIRTHWGHTT_NECIFIED), medical abortion (O15_ABORT_MEDICAL), preterm birth (O15_PRETERM), preterm birth (GCST008754), preterm birth (GCST008753), preterm birth (GCST90271753), preterm birth (GCST90271755), placental disorders (O15_PLAC_DISORD), preeclampsia (O15_PREECLAMPS), pregnancy hypertension (O15_HYPTENSPREG), pre-eclampsia or eclampsia (O15_PRE_OR_ECLAMPSIA), placenta previa (O15_PLAC_PREVIA), premature rupture of membranes (O15_MEMBR_PREMAT_RUPT), spontaneous abortion (O15_ABORT_SPONTAN) and spontaneous abortion (ukb-d-O03) respectively. APOs, adverse pregnancy outcomes.

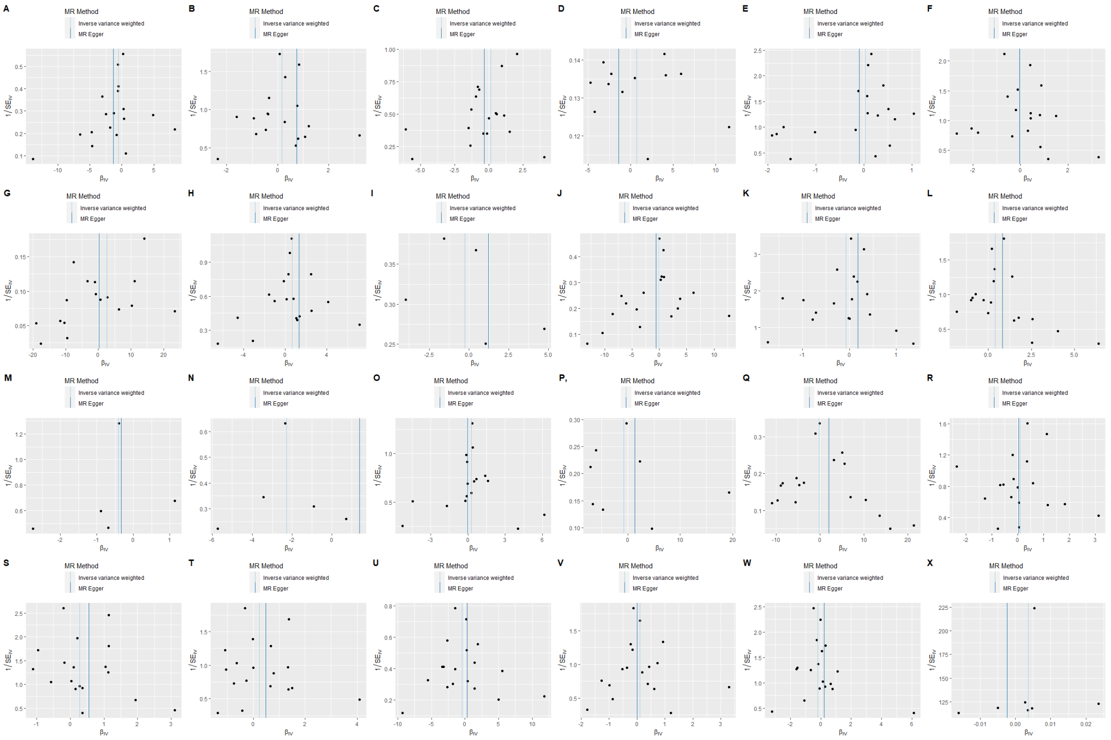

Figure S8. The funnel plots for of univariable mendelian randomization when exposure is the intake of artificial sweetener added to coffee. (A)-(X) refers the funnel plot of each APOs including abruption placenta (O15_PLAC_PREMAT_SEPAR), ectopic pregnancy (GCST90272883), excessive vomiting in pregnancy (O15_EXCESS_VOMIT_PREG), excessive vomiting in pregnancy (GCST90044480), gestational diabetes mellitus (GEST_DIABETES), gestational diabetes mellitus (GCST90296696), disorders associated with long gestation and high birth weight (R10_P16_DISORD_RELATED_LONG_GESTATION_HIGH_BIRTHWGHTT), intrahepatic cholestasis of pregnancy (O15_ICP_WIDE), intrahepatic cholestasis of pregnancy (GCST90095084), disorders related to short gestation and low birth weight (R10_P16_DISORD_RELATED_GESTATION_LOW_BIRTHWGHTT_NECIFIED), medical abortion (O15_ABORT_MEDICAL), preterm birth (O15_PRETERM), preterm birth (GCST008754), preterm birth (GCST008753), preterm birth (GCST90271753), preterm birth (GCST90271755), placental disorders (O15_PLAC_DISORD), preeclampsia (O15_PREECLAMPS), pregnancy hypertension (O15_HYPTENSPREG), pre-eclampsia or eclampsia (O15_PRE_OR_ECLAMPSIA), placenta previa (O15_PLAC_PREVIA), premature rupture of membranes (O15_MEMBR_PREMAT_RUPT), spontaneous abortion (O15_ABORT_SPONTAN) and spontaneous abortion (ukb-d-O03) respectively. APOs, adverse pregnancy outcomes.

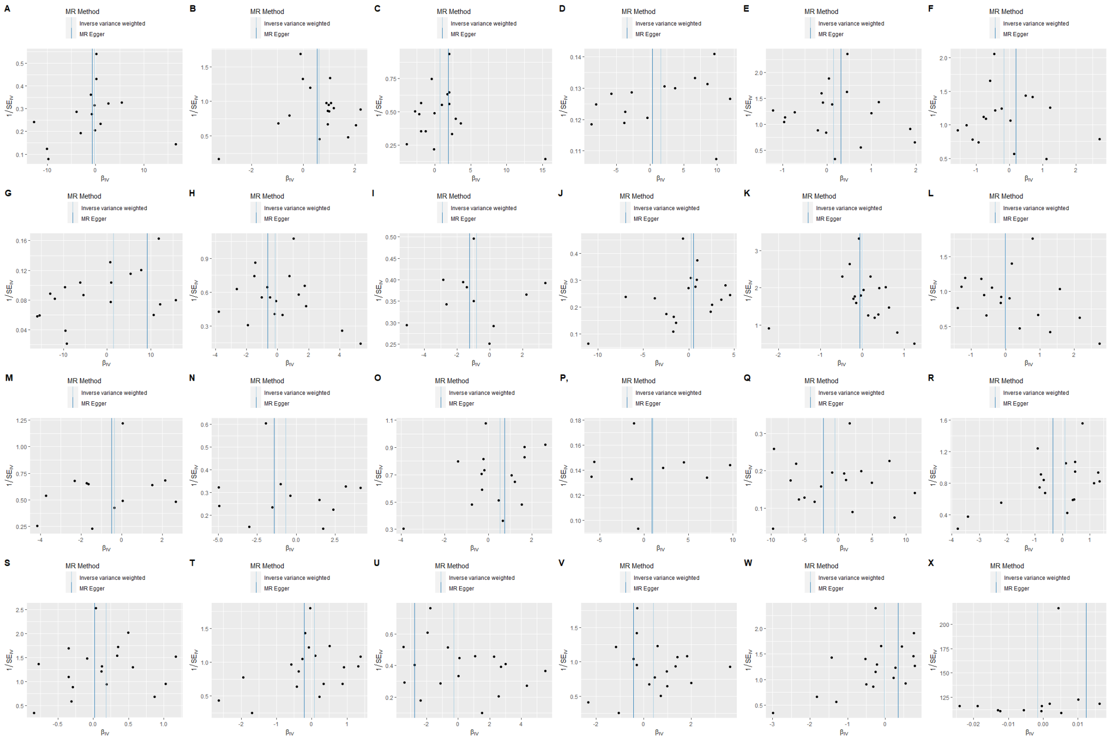

Figure S9. The funnel plots for of univariable mendelian randomization when exposure is the intake of artificial sweetener added to tea. (A)-(X) refers the funnel plot of each APOs including abruption placenta (O15_PLAC_PREMAT_SEPAR), ectopic pregnancy (GCST90272883), excessive vomiting in pregnancy (O15_EXCESS_VOMIT_PREG), excessive vomiting in pregnancy (GCST90044480), gestational diabetes mellitus (GEST_DIABETES), gestational diabetes mellitus (GCST90296696), disorders associated with long gestation and high birth weight (R10_P16_DISORD_RELATED_LONG_GESTATION_HIGH_BIRTHWGHTT), intrahepatic cholestasis of pregnancy (O15_ICP_WIDE), intrahepatic cholestasis of pregnancy (GCST90095084), disorders related to short gestation and low birth weight (R10_P16_DISORD_RELATED_GESTATION_LOW_BIRTHWGHTT_NECIFIED), medical abortion (O15_ABORT_MEDICAL), preterm birth (O15_PRETERM), preterm birth (GCST008754), preterm birth (GCST008753), preterm birth (GCST90271753), preterm birth (GCST90271755), placental disorders (O15_PLAC_DISORD), preeclampsia (O15_PREECLAMPS), pregnancy hypertension (O15_HYPTENSPREG), pre-eclampsia or eclampsia (O15_PRE_OR_ECLAMPSIA), placenta previa (O15_PLAC_PREVIA), premature rupture of membranes (O15_MEMBR_PREMAT_RUPT), spontaneous abortion (O15_ABORT_SPONTAN) and spontaneous abortion (ukb-d-O03) respectively. APOs, adverse pregnancy outcomes.

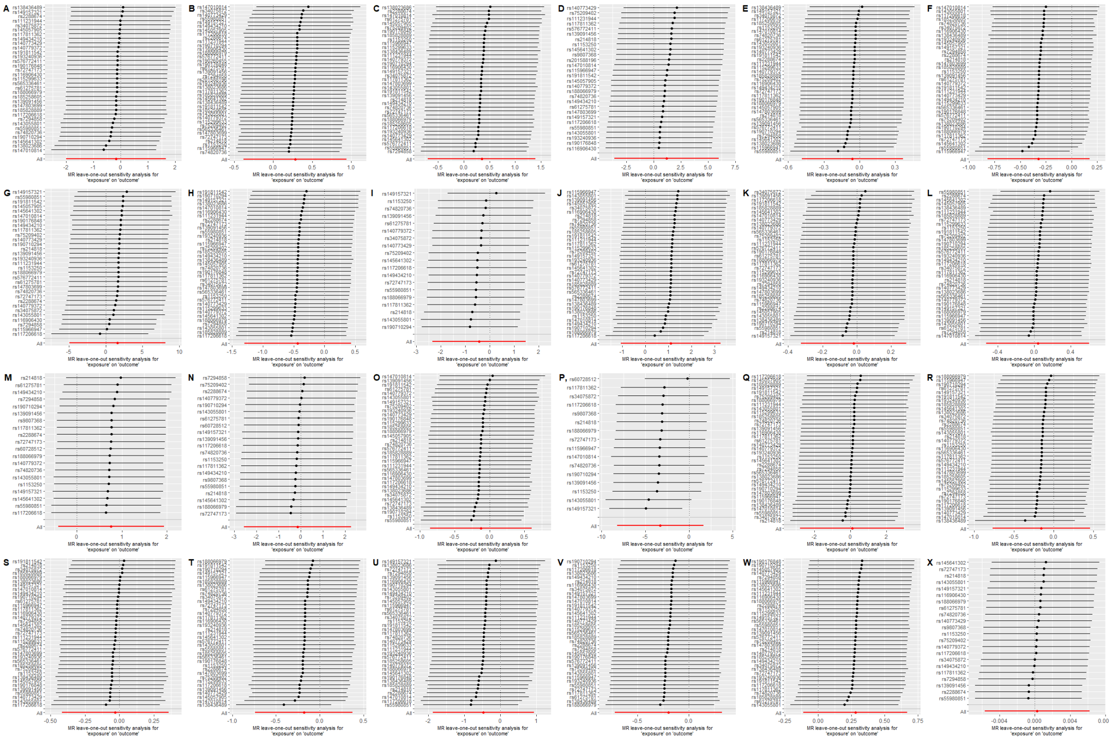

Figure S10. The results of ‘leave-one-out’ of univariable mendelian randomization when exposure is the intake of artificial sweetener added to cereal. (A)-(X) refers the ‘leave-one-out’ result of each APOs including abruption placenta (O15_PLAC_PREMAT_SEPAR), ectopic pregnancy (GCST90272883), excessive vomiting in pregnancy (O15_EXCESS_VOMIT_PREG), excessive vomiting in pregnancy (GCST90044480), gestational diabetes mellitus (GEST_DIABETES), gestational diabetes mellitus (GCST90296696), disorders associated with long gestation and high birth weight (R10_P16_DISORD_RELATED_LONG_GESTATION_HIGH_BIRTHWGHTT), intrahepatic cholestasis of pregnancy (O15_ICP_WIDE), intrahepatic cholestasis of pregnancy (GCST90095084), disorders related to short gestation and low birth weight (R10_P16_DISORD_RELATED_GESTATION_LOW_BIRTHWGHTT_NECIFIED), medical abortion (O15_ABORT_MEDICAL), preterm birth (O15_PRETERM), preterm birth (GCST008754), preterm birth (GCST008753), preterm birth (GCST90271753), preterm birth (GCST90271755), placental disorders (O15_PLAC_DISORD), preeclampsia (O15_PREECLAMPS), pregnancy hypertension (O15_HYPTENSPREG), pre-eclampsia or eclampsia (O15_PRE_OR_ECLAMPSIA), placenta previa (O15_PLAC_PREVIA), premature rupture of membranes (O15_MEMBR_PREMAT_RUPT), spontaneous abortion (O15_ABORT_SPONTAN) and spontaneous abortion (ukb-d-O03) respectively. APOs, adverse pregnancy outcomes.

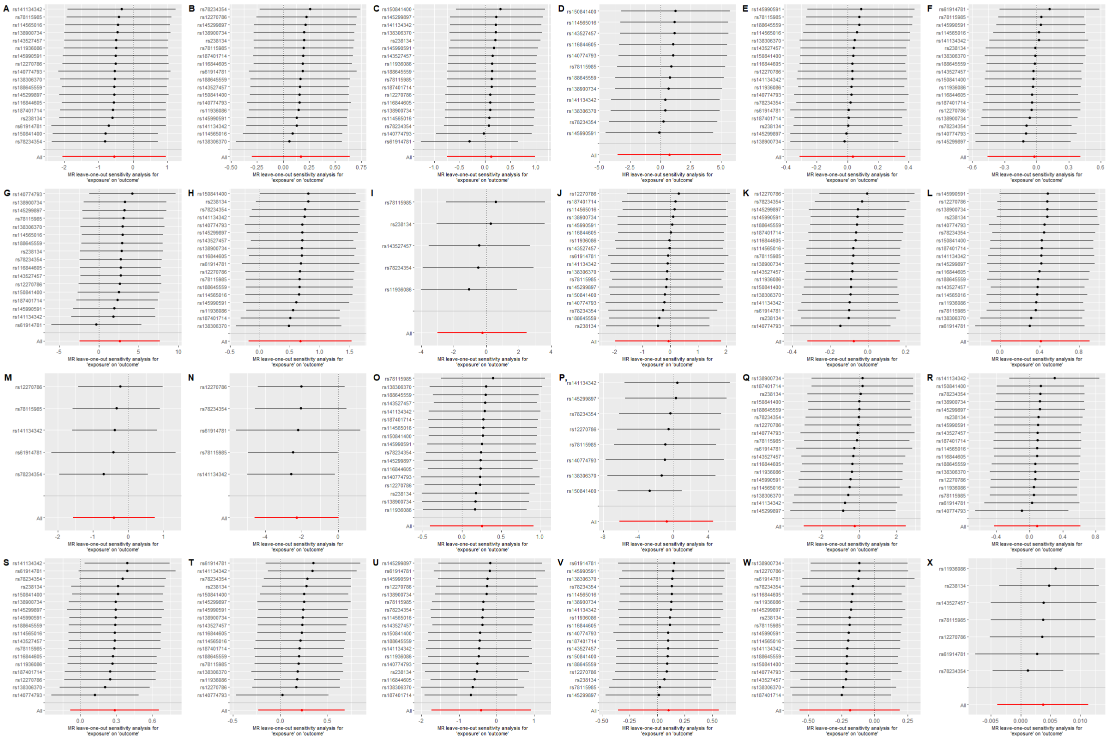

Figure S11. The results of 'leave-one-out' of univariable mendelian randomization when exposure is the intake of artificial sweetener added to coffee. (A)-(X) refers the 'leave-one-out' result of each APOs including abruption placenta (O15_PLAC_PREMAT_SEPAR), ectopic pregnancy (GCST90272883), excessive vomiting in pregnancy (O15_EXCESS_VOMIT_PREG), excessive vomiting in pregnancy (GCST90044480), gestational diabetes mellitus (GEST_DIABETES), gestational diabetes mellitus (GCST90296696), disorders associated with long gestation and high birth weight (R10_P16_DISORD_RELATED_LONG_GESTATION_HIGH_BIRTHWGHTT), intrahepatic cholestasis of pregnancy (O15_ICP_WIDE), intrahepatic cholestasis of pregnancy (GCST90095084), disorders related to short gestation and low birth weight (R10_P16_DISORD_RELATED_GESTATION_LOW_BIRTHWGHTT_NECIFIED), medical abortion (O15_ABORT_MEDICAL), preterm birth (O15_PRETERM), preterm birth (GCST008754), preterm birth (GCST008753), preterm birth (GCST90271753), preterm birth (GCST90271755), placental disorders (O15_PLAC_DISORD), preeclampsia (O15_PREECLAMPS), pregnancy hypertension (O15_HYPTENSPREG), pre-eclampsia or eclampsia (O15_PRE_OR_ECLAMPSIA), placenta previa (O15_PLAC_PREVIA), premature rupture of membranes (O15_MEMBR_PREMAT_RUPT), spontaneous abortion (O15_ABORT_SPONTAN) and spontaneous abortion (ukb-d-O03) respectively. APOs, adverse pregnancy outcomes.

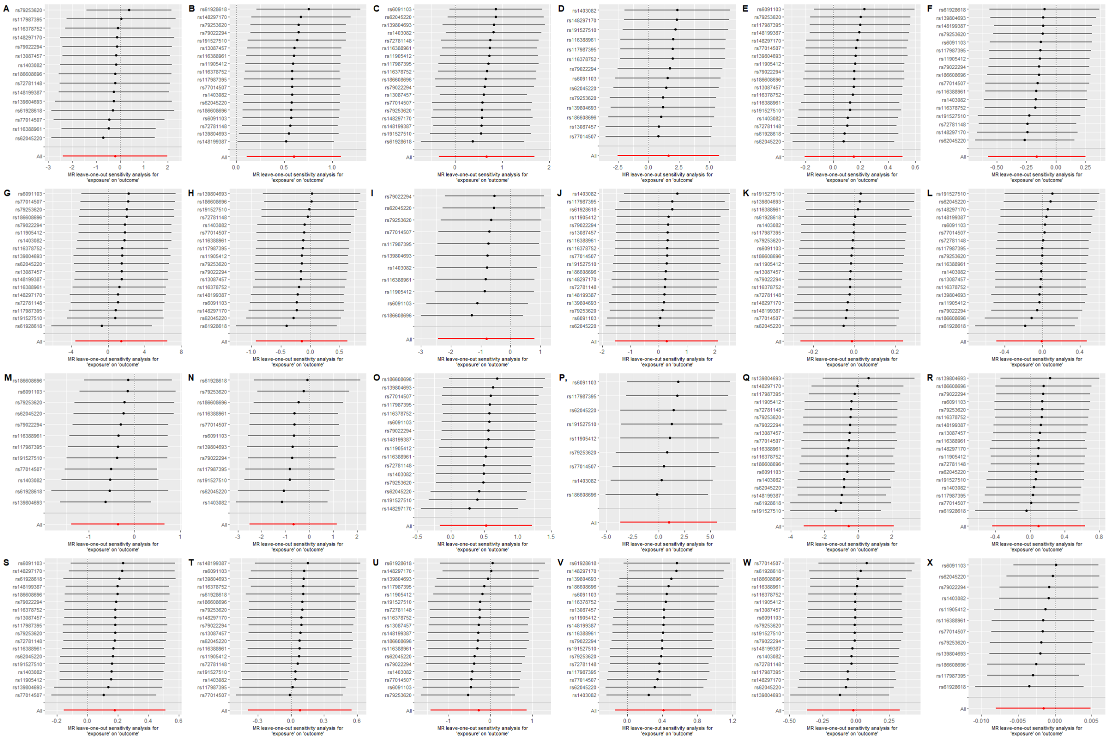

Figure S12. The results of 'leave-one-out' of univariable mendelian randomization when exposure is the intake of artificial sweetener added to tea. (A)-(X) refers the 'leave-one-out' result of each APOs including abruption placenta (O15_PLAC_PREMAT_SEPAR), ectopic pregnancy (GCST90272883), excessive vomiting in pregnancy (O15_EXCESS_VOMIT_PREG), excessive vomiting in pregnancy (GCST90044480), gestational diabetes mellitus (GEST_DIABETES), gestational diabetes mellitus (GCST90296696), disorders associated with long gestation and high birth weight (R10_P16_DISORD_RELATED_LONG_GESTATION_HIGH_BIRTHWGHTT), intrahepatic cholestasis of pregnancy (O15_ICP_WIDE), intrahepatic cholestasis of pregnancy (GCST90095084), disorders related to short gestation and low birth weight (R10_P16_DISORD_RELATED_GESTATION_LOW_BIRTHWGHTT_NECIFIED), medical abortion (O15_ABORT_MEDICAL), preterm birth (O15_PRETERM), preterm birth (GCST008754), preterm birth (GCST008753), preterm birth (GCST90271753), preterm birth (GCST90271755), placental disorders (O15_PLAC_DISORD), preeclampsia (O15_PREECLAMPS), pregnancy hypertension (O15_HYPTENSPREG), pre-eclampsia or eclampsia (O15_PRE_OR_ECLAMPSIA), placenta previa (O15_PLAC_PREVIA), premature rupture of membranes (O15_MEMBR_PREMAT_RUPT), spontaneous abortion (O15_ABORT_SPONTAN) and spontaneous abortion (ukb-d-O03) respectively. APOs, adverse pregnancy outcomes.
